# A Model for NAD(P)H:Quinoneoxidoreductase 1 (NQO1) Targeted Individualized Cancer Chemotherapy

**DOI:** 10.4137/dti.s1146

**Published:** 2009-01-15

**Authors:** Asher Begleiter, Nadia El-Gabalawy, Laurie Lange, Marsha K. Leith, Lynn J. Guziec, Frank S. Guziec

**Affiliations:** 1 Manitoba Institute of Cell Biology, CancerCare Manitoba, Departments of Internal Medicine and Pharmacology and Therapeutics, University of Manitoba, 675 McDermot Avenue, Winnipeg, Manitoba R3E 0V9 Canada; 2 Manitoba Institute of Cell Biology, CancerCare Manitoba, 675 McDermot Avenue, Winnipeg, Manitoba R3E 0V9 Canada; 3 Department of Chemistry and Biochemistry, Southwestern University, Georgetown, Texas 78628 U.S.A

**Keywords:** individualized cancer chemotherapy, tumor targeting, bioreductive agents, NQO1, benzoquinone mustard

## Abstract

NQO1 (NAD(P)H:quinoneoxidoreductase 1) is a reductive enzyme that is an important activator of bioreductive antitumor agents. NQO1 activity varies in individual tumors but is generally higher in tumor cells than in normal cells. NQO1 has been used as a target for tumor specific drug development. We investigated a series of bioreductive benzoquinone mustard analogs as a model for NQO1 targeted individualized cancer chemotherapy. We compared the tumor cell growth inhibitory activity of benzoquinone mustard analogs with sterically bulky groups of different size and placed at different positions on the benzoquinone ring, using tumor cell lines with different levels of NQO1. We demonstrated that functional groups of different steric size could be used to produce a series of bioreductive antitumor agents that were activated by different levels of NQO1 in tumor cells. This series of drugs could then be used to target cells with specific levels of NQO1 for growth inhibition and to avoid damage to normal cells, like bone marrow cells, that have low levels of NQO1. This approach could be used to develop new bioreductive antitumor agents for NQO1 targeted individualized cancer chemotherapy.

## Introduction

Current anticancer agents are generally potent enough to kill most cancer cells, but their use is limited by toxic side effects. Similarities between cancer and normal cells result in a narrow therapeutic index. The use of chemotherapy could be greatly enhanced by new drugs that specifically target cancer cells. An important strategy to improve selectivity of anticancer drugs for cancer cells is enzyme-directed tumor targeting.^1^,^2^ In this strategy drugs activated by a specific enzyme are used to treat tumors with high levels of that enzyme.

Bioreductive agents are a class of anticancer drugs that must be activated in cells by reductive enzymes. These agents are preferentially active in solid tumors, which represent the majority of cancers and produce the highest mortality. The clinical potential^3^,^4^ and mode of action^5^,^6^ of these agents have been actively studied, and the use of the prototype drug mitomycin C (MMC) in the clinic has been reviewed.^7^ Other agents like porfiromycin, diaziquone (AZQ), 3-hydroxymethyl-5-aziridinyl-1-methyl-2-(1*H*-indole-4,7-dione)prop-β-en-α-ol (EO9), tirapazamine (TPZ) and 2,5-diaziridinyl-3-(hydroxymethyl)-6-methyl-1,4-benzoquinone (RH1) have been tested clinically,^7^–^15^ and there is also ongoing work to develop new agents.^16^,^17^ These drugs are potent antitumor agents, but produce a variety of side effects including lung,^7^,^18^ hearing, muscle and renal toxicity.^10^,^12^ However, marrow toxicity is often the dose limiting toxicity for these agents.^7^,^9^,^10^,^19^ Bioreductive agents can be activated by two electron reducing enzymes like NAD(P)H:quinoneoxidoreductase 1 (NQO1; DT-diaphorase)^1^,^20^ or one electron reducing enzymes like NADPH cytochrome P450 reductase (P450 Red).^5^,^21^

NQO1 is a flavoprotein that catalyzes 2-electron reduction of quinones and N-oxides.^1^ Several human diaphorases are known,^1^,^22^,^23^ but NQO1 appears to be most important for activating bioreductive agents.^1^,^22^,^24^ NQO1 is a homodimer that uses NAD(P)H as an electron donor.^1^ The enzyme is mainly cytosolic, but a significant proportion of the enzyme is present in the nucleus of cancer cells.^25^ It is ubiquitous in eukaryotes but levels vary in different tissues,^1^,^24^,^26^ with low levels in hematopoetic cells.^26^ Activity is usually higher in tumor than normal cells.^24^,^26^,^26^

NQO1 has been shown to play a role in the activation of cancer chemotherapeutic agents,^1^,^20^,^28^ detoxification of xenobiotics^29^ and cancer prevention.^20^,^28^ Activation of bioreductive agents by NQO1 has been extensively studied.^1^,^20^ Cells with elevated NQO1 levels are more sensitive to MMC and drug activity is decreased by the NQO1 inhibitor, dicoumarol.^30^–^32^ Thus, NQO1 is a major activating enzyme for MMC. NQO1 is also the major activating enzyme for EO9, 2,5-diaziridinyl-3,6-dimethyl-1,4-benzoquinone (MeDZQ) and RH1.^1^,^4^,^14^,^33^,^34^

Bioreductive agents are ideally suited for enzyme-directed tumor targeting because they must be activated in cells by reductive enzymes.^1^,^2^ NQO1 has been extensively used as a target for this strategy because it is found in all tissues,^24^,^26^ and is preferentially expressed in tumor cells.^24^,^26^,^27^ In addition, NQO1 levels are generally low in hematopoetic cells.^26^ Thus, agents, like RH1, that are selectively activated by NQO1,^14^ have been developed to target tumors with high NQO1 levels and to avoid bone marrow toxicity.

Current bioreductive agents have demonstrated the potential of this strategy for improved cancer chemotherapy; however, a number of problems have hindered the development of new targeted anticancer agents. To use NQO1 as a target for enzyme-directed tumor targeting requires an agent specifically activated by NQO1. If the drug is also activated by P450 Red, which is found at appreciable levels in hematopoetic cells and other organs, this may produce toxic side effects as is seen with MMC.^7^ However, NQO1 levels vary in individual human tumors. Thus, antitumor agents that have a high affinity for NQO1 may be activated by the low levels of this enzyme in marrow cells resulting in marrow toxicity, while agents that have a low affinity for NQO1 may not be fully activated in tumors with lower levels of this enzyme resulting in a poor tumor response. The former situation is illustrated by the observation that RH1, which is highly specific for activation by NQO1^14^ but has very high affinity for this enzyme,^35^ produces significant marrow toxicity.^19^ An example of the latter situation is the resistance to MMC observed in tumor cells with low levels of NQO1.^30^,^36^,^37^

Bioreductive agents need a bioreductive element that is reduced and a cytotoxic element that is activated by this reduction and produces the cytotoxic effects. Current bioreductive agents generally use quinones or nitrogen-oxides as bioreductive elements and alkylating groups like aziridines or nitrogen mustards as cytotoxic elements. Alkylating groups bind to DNA to produce DNA crosslinks^1^,^38^,^39^ leading to cell death by apoptosis.^40^ Using a series of benzoquinone mustards (BM) analogs, we found that sterically bulky groups on the benzoquinone ring significantly decreased the rate of reduction of the BM analogs by NQO1 and reduced their cytotoxic and DNA crosslinking activities.^41^,^42^ This was likely due to interference by the bulky groups with the ability of the benzoquinone to fit into the active site of NQO1. The effect of these bulky groups was also influenced by the position of the group on the benzoquinone ring moiety. Thus, adding an appropriate sterically bulky group to the benzoquinone structure could decrease the affinity for NQO1. This would make it more difficult to activate the new agent in cells with very low levels of NQO1, like bone marrow cells, but could still allow activation in tumor cells with higher levels of this enzyme. Since the level of NQO1 in tumors varies in different individuals, an anticancer agent with the minimum affinity for NQO1 to be fully activated by the level of enzyme in the tumor of a patient would provide the maximum antitumor effect, while minimizing activation in the marrow and the resulting marrow toxicity for that individual. This strategy could provide an optimized therapeutic index for each patient.

In this study, we investigated a series of BM analogs as a model for NQO1 targeted individualized cancer chemotherapy. We compared the tumor cell growth inhibitory activity of BM analogs with sterically bulky groups of different size and placed at different positions on the benzoquinone ring, using tumor cell lines with different levels of NQO1. We demonstrated that it was possible to design bioreductive agents with the appropriate NQO1 affinity to target tumors with specific levels of NQO1.

## Materials and Methods

All media, fetal bovine serum and Hank’s balanced salt solutions were obtained from Invitrogen (Burlington ON). All reagents for the NQO1 activity and MTT assays were obtained from Sigma-Aldrich (St. Louis, MO). The FaDu, HCT116 and T47D cell lines were obtained from the American Type Culture Collection (Rockville, MD). FaDu, human pharynx squamous carcinoma cells, HCT116, human colon carcinoma cells and T47D, human breast ductal carcinoma cells were grown in Dulbecco’s modified Eagle’s medium: Hams F12 (1:1) media with 10% fetal bovine serum.

**Figure 1.**
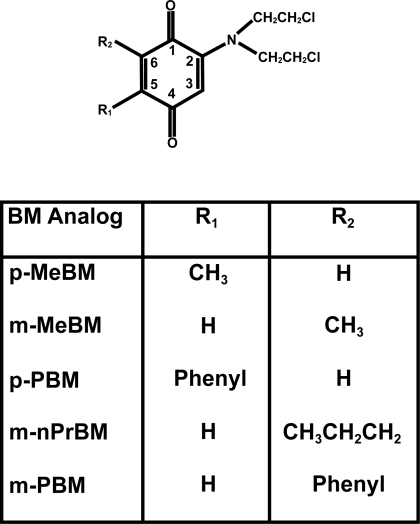
Structures of BM analogs.

The syntheses of the BM analogs p-MeBM, m-MeBM, p-PBM and m-PBM (Fig. 1) have been previously reported.^41^,^42^ m-nPrBM was synthesized as described below.

For the preparation of the m-nPrBM precursor 2-n-propylbenzoquinone, benzoquinone (5.184 g, 48.0 mmol), butyric acid (3.524 g, 40.0 mmol), and silver nitrate (1.02 g, 6.0 mmol) in a acetonitrile-water mixture (1:1) (200 mL) were heated to reflux while a solution of 1.0 M ammonium persulfate (48 mL) was added dropwise over 1 h. After an additional 1.25 h reflux, the mixture was cooled to room temperature and solid sodium bicarbonate (3.36 g, 40 mmol) was added in portions to neutralize excess butyric acid. The mixture was extracted with ethyl ether (3x100 mL) and the ether phase washed with 0.5 M sodium bicarbonate, brine and dried over anhydrous sodium sulfate. The crude mixture was concentrated and dissolved in hexanes-ethyl acetate (7:1) (30 mL), and filtered through a short column of (50 g) silica gel. Concentration afforded the crude product as a yellow oil, 3.51 g. A portion of crude mixture (665 mg) was chromatographed on silica using hexanes-ethyl acetate (7:1), affording pure 2-n-propylbenzoquinone (229 mg) as a yellow semi-solid in 24% overall yield. NMR (CDCl_3_): δ 6.69–6.78 (complex, 2H), 6.57 (m, 1H), 2.40 (t, 2H), 1.55 (sextet, 2H), 0.98 (t, 3H).

For preparation of m-nPrBM 6-n-propyl-2-[bis(2-chloroethylamino)]-1,4-benzoquinone (Fig. 1), potassium fluoride (0.266 g, 4.58 mmol) was added at room temperature to a stirred suspension of 2-n-propylbenzoquinone (0.229 g, 1.53 mmol), bis(2-chloroethylamine) hydrochloride (0.817 g, 4.58 mmol) and cupric acetate (0.187 g, 2.29 mmol) in 95% ethanol (6.0 mL). The mixture was stirred open to the air but protected from light for 69 h. The mixture was filtered with suction through a Celite pad to remove copper salts and the precipitate washed with ethyl acetate (2 × 10 mL). The filtrates were concentrated, and the residue was taken up in ethyl acetate (30 mL). The organic phase was washed with 0.15 M HCl (15 mL), brine, and then dried over anhydrous sodium sulfate. Concentration, followed by silica chromatography using ethyl acetate-hexanes (1:2) afforded the 6-n-propyl-2-[bis(2-chloroethylamino)]-1,4-benzoqui-none as a red semi-solid, 0.440 g, 10% yield. ^1^H NMR (CDCl_3_): δ 6.42 (m, 1H), 5.63 (d, 1H, J = 2.4 Hz), 3.82–3.78 (m, 4H), 3.74–3.69 (m, 4H), 2.37 (t, 2H), 1.54 (sextet, 2H), 0.97 (t, 3H); ^13^C NMR (CDCl_3_): δ 186.0, 185.6, 149.0, 147.5, 133.0, 106.0, 54.8, 40.4, 31.2, 21.1, 13.8.

NQO1 activity in the cell lines was determined as we have previously described^43^ using 2, 6-dichlorophenolindophenol (DCPIP) as the electron acceptor. The statistical significance of differences in the mean values of the NQO1 activities in the three cell lines was evaluated using a one-way analysis of variance.

For cell growth inhibition studies, BM analogs were prepared at various concentrations in dimethylformamide and were added to cells in media. The final concentration of dimethylformamide in the cells was 1%. The cells were incubated with the BM analog at 37 ºC for 1 h. The cells were washed and incubated for 4 cell doublings at 37 °C. Cell growth inhibition was determined by 3-(4, 5-dimethylthiazo-2-yl)-2,5-diphenyltetrazolium bromide (MTT) assay as we have previously described.^44^,^45^ The results are presented as relative absorbance compared with control *vs* drug concentration curves, and as IC_50_ values (concentration of drug reducing the relative absorbance to 0.5) obtained from the linear regression lines of the relative absorbance *vs* drug concentration curves for each BM analog. The IC_50_ values for the 5 BM analogs in each cell line were compared by one way analysis of variance with Student-Newman-Kuels pairwise multiple comparison.

## Results

### Description of the BM analogs and cell lines

The synthesis of the benzoquinone mustards, p-MeBM, m-MeBM, p-PBM and m-PBM was previously reported.^41^,^42^ Here we report the synthesis of a new BM analog, m-nPrBM (Fig. 1). p-MeBM and p-PBM have functional group substitutions at the C5 position of the benzoquinone ring, while m-MeBM, m-nPrBM and m-PBM have functional group substitution at the C6 position of the ring. The relative steric size of the functional groups is CH_3_ < CH_3_CH_2_CH_2_ < phenyl. The tumor cell growth inhibitory effects of the BM analogs in human cancer cells with different levels of NQO1 were studied to determine if the size and position of the functional groups could determine the level of NQO1 required to activate the analogs.

**Figure 2.**
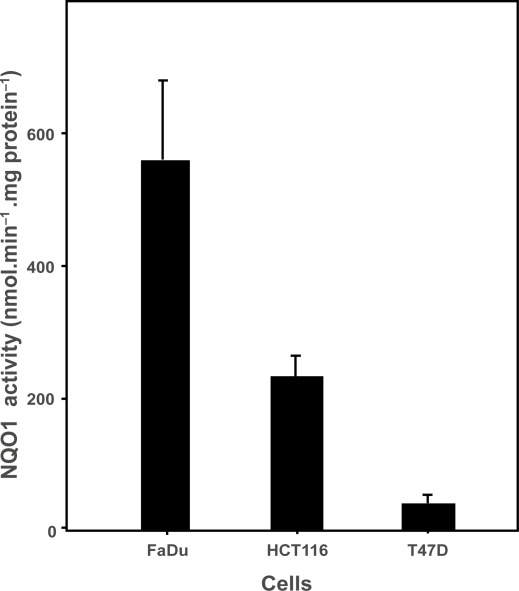
NQO1 activity in tumor cell lines. NQO1 activities in FaDu, HCT116 and T47D cells were determined as described.^43^ Bars represent the mean ± SEM of 3 or 4 determinations.

Tumor cell growth inhibition studies were carried out in FaDu human pharynx squamous carcinoma cells, HCT116 human colon carcinoma cells or T47D human breast carcinoma cells. The mean ± standard error of the mean (SEM) NQO1 activity in these cells was 557.6 ± 120.3, 230.4 ± 31.3 and 39.7 ± 12.1 nmol.min^−1^.mg protein^−1^, respectively, and these activities were statistically different (p < 0.02) (Fig. 2).

### Tumor cell growth inhibition by BM analogs

**Figure 3.**
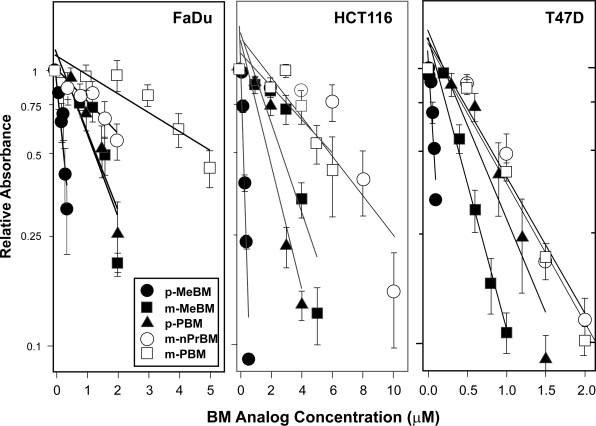
Cell growth inhibition produced by BM analogs in FaDu, HCT116 and T47D cells. Cells were incubated at 37 °C for 1 h with various concentrations of each BM analog and cell growth inhibition was determined by MTT assay as we have previously described.^44^,^45^ The results are presented as relative absorbance compared with control *vs.* drug concentration. Points represent the mean ± SEM of 3 or 4 determinations. The lines are linear regression lines.

FaDu, HCT116 or T47D cells were treated with various concentrations of each BM analog for 1 h and tumor cell growth inhibition was determined by MTT assay.^44^,^45^ Figure 3 shows the growth inhibition curves for each of the 5 BM analogs in the 3 tumor cell lines. In all three cell lines, p-MeBM had the greatest inhibitory effect while m-PBM had the smallest effect. In FaDu cells, the other 3 BM analogs produced intermediate effects. In HCT116 cells, m-MeBM and p-PBM continued to produce an intermediate inhibitory effect; however, tumor cell growth inhibition by m-nPrBM was lower and was similar to that produced by m-PBM. In T47D cells, m-MeBM again produced an intermediate tumor cell growth inhibitory effect, while p-PBM, m-nPrBM and m-PBM all produced lower but similar inhibitory effects.

Table 1 shows the IC_50_ values for the tumor cell growth inhibitory effects of the BM analogs in FaDU, HCT116 and T47D cells. There were differences in the overall sensitivity of the cell lines to the BM analogs; however, there were also specific differences in the sensitivities of the cell lines to individual drugs. In FaDu cells, the relative tumor cell growth inhibitory effects of the BM analogs were: p-MeBM > m-MeBM = p-PBM > m-nPrBM > m-PBM. In HCT116 cells, the relative inhibitory effects were: p-MeBM > m-MeBM = p-PBM > m-nPrBM = m-PBM. While in T47D cells, the relative inhibitory effects were: p-MeBM > m-MeBM > p-PBM = m-nPrBM = m-PBM. The differences in activities of the different analogs in each cell line were statistically significant by one way analysis of variance (p < 0.001).

## Discussion

We have previously demonstrated that functional groups can significantly influence the reduction and activation of BM bioreductive agents by NQO1.^41^,^42^ Activation of these agents was decreased as the steric size of the functional group increased, and this affect was greater when the functional group was at the C6 position on the benzoquinone ring compared with the C5 position of the ring. We made use of these findings to design a series of BM analogs with different affinities for activation by NQO1, as a model for developing NQO1 targeted individualized cancer chemotherapy. The analogs p-MeBM and m-MeBM have small methyl functional groups at C5 and C6 of the benzoquinone ring, respectively. We expected these analogs to be most easily activated by NQO1 with p-MeBM being activated by a lower level of NQO1 activity than m-MeBM. The p-PBM and m-PBM analogs have larger phenyl functional groups at C5 and C6 of the ring, respectively. We expected these analogs to be less easily activated by NQO1 than the methyl analogs, with m-PBM requiring the highest level of NQO1 activity. The m-nPrBM analog has an n-propyl functional group at C6 of the benzoquinone ring. This group is smaller than the phenyl group and should produce a BM analog that is activated by intermediate levels of NQO1.

Human tumor cells have varied levels of NQO1 activity ranging from < 10 nmol. min^−1^.mg protein^−1^ to > 1000 nmol. min^−1^.mg protein^−1^. In contrast, human bone marrow cells generally have a level of NQO1 activity < 30 nmol. min^−1^.mg protein^−1^. Since bone marrow toxicity is often the dose limiting toxicity for bioreductive agents, an agent that is fully activated by the level of NQO1 in the tumor but is not activated by the level of NQO1 in bone marrow cells would produce little bone marrow toxicity and would have the highest therapeutic index. If a series of bioreductive agents that were activated by different levels of NQO1 were available, a patient could be treated with an agent that was maximally activated by the level of NQO1 in their tumor but minimally activated by the level of NQO1 in their bone marrow.

To test this hypothesis, we measured tumor cell growth inhibition produced by the series of BM analogs having different affinities for activation by NQO1, in human tumor cell lines with a wide range of NQO1 activity. FaDu human pharynx squamous carcinoma cells had a high level of NQO1 activity, 557.6 ± 120.3 nmol.min^−1^.mg protein^−1^, while HCT116 human colon carcinoma cells had a level of NQO1 activity that was close to the average for human tumors, 230.4 ± 31 nmol.min^−1^.mg protein^−1^ (Fig. 2). In contrast, T47D human breast carcinoma cells had a low level of NQO1 activity similar to that found in human bone marrow cells, 39.7 ± 12.1 nmol.min^−1^.mg protein^−1^.

When we compared the inhibition of tumor cell growth produced by the BM analogs in the three tumor cell lines we observed a number of significant differences. Looking at the overall activities of the BM analogs, the FaDu and HCT116 cells appeared to have similar sensitivities to the BM analogs as a group, with IC_50_ values ranging from approximately 0.25 to 6.00 μM in both cell lines. In contrast, the T47D cells appeared to be overall approximately 5-fold more sensitive to these agents as a group (Fig. 3, Table 1). These differences likely reflect general tissue type and/or cell line variations in sensitivity to these agents due to differences in drug uptake, drug detoxification, DNA repair, induction of apoptosis and activation by other enzymes, and are probably not related to activation of the analogs by NQO1. This suggestion is supported by the finding that in relative terms, p-MeBM had the largest growth inhibitory effect; m-PBM had the smallest inhibitory effect, and the other BM analogs had intermediate inhibitory effects in all three cell lines.

When we examined the growth inhibitory activities of the five BM analogs within each cell line, we observed significant differences in the relative activity of the analogs in the different cell lines. In the FaDu cells with the highest NQO1 activity, the relative tumor cell growth inhibitory activity was p-MeBM > m-MeBM = p-PBM > m-nPrBM = m-PBM. This suggests that p-MeBM was most activated; m-MeBM and p-PBM were substantially activated; m-nPrBM was somewhat activated, and m-PBM was poorly activated by the level of NQO1 in these cells. This is consistent with our hypothesis based on the steric size and location of the functional groups. In the HCT116 cells with an intermediate level of NQO1 activity, the relative inhibitory activity was p-MeBM > m-MeBM = p-PBM > m-nPrBM = m-PBM, suggesting that p-MeBM was most activated; m-MeBM and p-PBM were substantially activated, and m-nPrBM and m-PBM were poorly activated by the level of NQO1 in these cells. This represents a decrease in the relative growth inhibitory effect of m-nPrBM in the HCT116 cells compared with the FaDu cells. This suggests that the n-propyl functional group at the C6 position produces a greater steric effect than the methyl group at the C6 position and the phenyl group at the C5 position. In addition, this result suggests that m-nPrBM would not be fully activated in tumors with intermediate levels of NQO1 and would only be maximally effective in tumors with high levels of NQO1 activity. In T47D cells with the lowest NQO1 activity, the relative growth inhibitory activity was p-MeBM > m-MeBM > p-PBM = m-nPrBM = m-PBM, suggesting that p-MeBM was most activated; m-MeBM was substantially activated, and p-PBM, m-nPrBM and m-PBM were poorly activated by the level of NQO1 in these cells. This represents a decrease in the relative growth inhibitory effect of p-PBM in T47D cells compared with HCT116 cells. This result suggests that p-PBM would not be fully activated in tumors with low levels of NQO1 and would only be effective in tumors with average or higher NQO1 activity. Furthermore, these findings indicate that m-MeBM and p-MeBM are at least partially activated in cells with low levels of NQO1 activity like bone marrow cells, and would likely produce some bone marrow toxicity.

Since m-nPrBM and p-PBM showed good tumor cell growth inhibition in the FaDu cells but poor activity in the T47D cells, these BM analogs would likely have good antitumor activity in tumors with high NQO1 activity but little activity in bone marrow cells. Because p-PBM also showed good growth inhibition in HCT116 cells, it would likely also have good antitumor activity in tumors with average levels of NQO1, again with little bone marrow toxicity. Thus, m-nPrBM or p-PBM might be suitable agents for use in patients having tumors with high NQO1; p-PBM might be suitable for use in patients having tumors with average NQO1, and m-MeBM might be used in patients having tumors with low NQO1. However, the use of m-MeBM would likely result in at least some bone marrow toxicity. The use of p-MeBM would likely produce significant bone marrow toxicity in most patients. These results demonstrate the potential of using a series of bioreductive agents with different affinities for activation by NQO1 for targeted individualized cancer chemotherapy. They also suggest that using functional groups of varying steric size with quinone based bioreductive agents represents a feasible approach to designing new agents with a range of affinities for activation by NQO1.

In summary this study has demonstrated that functional groups of different steric size can be used to produce a series of bioreductive antitumor agents that are activated by different levels of NQO1 in tumor cells. A series of drugs of this type might be used to target cells with specific levels of NQO1 for growth inhibition and to avoid damage to normal cells, like bone marrow cells, that have low levels of NQO1. This approach could be used to develop new bioreductive antitumor agents for NQO1 targeted individualized cancer chemotherapy that produce the optimal therapeutic index for each patient.

## Figures and Tables

**Table 1.** Tumor cell growth inhibition by BM analogs in cell lines with different NQO1 activities.

**BM analog**	**IC_50_(μM)**		
	**FaDu (n)^1^**	**HCT116 (n)^1^**	**T47D (n)^1^**
p-MeBM	0.30 ± 0.08 (4)	0.22 ± 0.01 (3)	0.08 ± 0.01 (4)
m-MeBM	1.27 ± 0.13 (4)	2.69 ± 0.25 (4)	0.39 ± 0.04 (4)
p-PBM	1.39 ± 0.31 (4)	1.85 ± 0.13 (3)	0.63 ± 0.08 (4)
m-nPrBM	3.10 ± 0.95 (4)	6.10 ± 1.18 (4)	0.80 ± 0.08 (4)
m-PBM	5.77 ± 1.31 (4)	6.68 ± 1.53 (3)	0.76 ± 0.02 (3)

1 Mean ± SEM.

n = number of determinations.
